# Efficacy of Auditory Training in Elderly Subjects

**DOI:** 10.3389/fnagi.2015.00078

**Published:** 2015-05-18

**Authors:** Aline Albuquerque Morais, Caroline Nunes Rocha-Muniz, Eliane Schochat

**Affiliations:** ^1^Auditory Processing Laboratory, Department of Physical Therapy, Speech Therapy and Occupational Therapy, University of São Paulo, São Paulo, Brazil

**Keywords:** auditory perception, auditory processing disorder, auditory training, elderly, speech comprehension, aging, neuroplasticity, P300 event-related potential

## Abstract

Auditory training (AT) has been used for auditory rehabilitation in elderly individuals and is an effective tool for optimizing speech processing in this population. However, it is necessary to distinguish training-related improvements from placebo and test–retest effects. Thus, we investigated the efficacy of short-term AT [acoustically controlled auditory training (ACAT)] in elderly subjects through behavioral measures and P300. Sixteen elderly individuals with auditory processing disorder (APD) received an initial evaluation (evaluation 1 – E1) consisting of behavioral and electrophysiological tests (P300 evoked by tone burst and speech sounds) to evaluate their auditory processing. The individuals were divided into two groups. The Active Control Group (*n* = 8) underwent placebo training. The Passive Control Group (*n* = 8) did not receive any intervention. After 12 weeks, the subjects were revaluated (evaluation 2 – E2). Then, all of the subjects underwent ACAT. Following another 12 weeks (eight training sessions), they underwent the final evaluation (evaluation 3 – E3). There was no significant difference between E1 and E2 in the behavioral test [*F*(9.6) = 0.06, *p* = 0.92, λ de Wilks = 0.65)] or P300 [*F*(8.7) = 2.11, *p* = 0.17, λ de Wilks = 0.29] (discarding the presence of placebo effects and test–retest). A significant improvement was observed between the pre- and post-ACAT conditions (E2 and E3) for all auditory skills according to the behavioral methods [*F*(4.27) = 0.18, *p* = 0.94, λ de Wilks = 0.97]. However, the same result was not observed for P300 in any condition. There was no significant difference between P300 stimuli. The ACAT improved the behavioral performance of the elderly for all auditory skills and was an effective method for hearing rehabilitation.

## Introduction

The structural and functional changes in the auditory system due to aging can limit speech comprehension during difficult listening situations in elderly people (Corso, 1977; Jerger et al., 1989; Willott, 1991; Chisolm et al., 2003; Gates and Mills, 2005). Previous studies have demonstrated poor performance of elderly people compared with young people during different auditory tasks, including temporal processing, listening in noisy environments, and dichotic listening, indicating that the difficulty in understanding speech among elderly people may be associated with auditory processing disorder (APD) (Dubno et al., 1984; Welsh et al., 1985; Jerger et al., 1989; Cooper and Gates, 1991; Snell, 1997; Phillips et al., 2000; Bellis and Wilber, 2001; Pichora-Fuller and Souza, 2003; Gates and Mills, 2005; Martin and Jerger, 2005; Kraus and Anderson, 2013). Executive functions, such as short-term memory, attention, inhibition, and decision-making, are also essential for understanding speech (Humes, 1996; Pichora-Fuller, 2003). Therefore, auditory rehabilitation in elderly people should include actions to minimize peripheral hearing losses and central auditory and executive skills.

Previous studies on auditory training (AT) have demonstrated favorable results in auditory and cognitive perception among elderly people (consequently improving their social participation and quality of life) (Pichora-Fuller and Souza, 2003; Smith et al., 2009; Anderson et al., 2013a,b; Ferguson et al., 2014). AT is based on the plasticity of the central nervous system (Chermak and Musiek, 2002). Previously reported methods have used different tasks, such as more sensory or more cognitive methods; methods performed in a soundproof booth; utilizing specific software programs; methods performed in the home; and different frequencies and durations. Despite the heterogeneity of these methods, AT is generally an effective tool for the auditory rehabilitation in adults (Sweetow and Palmer, 2005; Pichora-Fuller and Levitt, 2012; Henshaw and Ferguson, 2013).

Although AT induces improvements in auditory processing, the effects of short-term training on the aging auditory nervous system remain unknown. Furthermore, according to Ferguson et al. (2014), it is not entirely clear how this intervention can provide real improvements in auditory skills (despite the large increase in the number of products and studies on AT). According to these authors, few studies have used untrained control groups and/or groups undergoing placebo training. The lack of controls may lead to ambiguous data interpretation. Placebo and test–retest effects cannot be ignored. Therefore, it is essential that interventional studies involving AT consider the possibility of these effects when analyzing the results.

Both behavioral and electrophysiological tests of auditory processing have been widely used to monitor auditory interventions. Significant changes in bioelectrical activity within the auditory system are observed after AT (Jirsa, 1992; Kraus et al., 1995; Tremblay et al., 1997, 2001).

P300 is a long latency potential that occurs approximately 300 ms after a stimulus presentation. It can be elicited by the oddball paradigm, which involves the detection and discrimination of a rare stimulus amid a series of frequent stimuli. P300 can be influenced by higher cognitive functions, including attention and memory, and originates in the primary and secondary areas of the cortex. However, the exact elicitors are unknown (McPherson, 1996; Linden et al., 1999; Musiek and Lee, 2001; Schochat, 2003; Linden, 2005; Polich, 2007). Therefore, this study was motivated by the need to investigate the effects of short-term training [termed acoustically controlled auditory training (ACAT)] on the aging auditory system using untrained control groups and/or training in placebo groups. This study investigated the effectiveness of ACAT in the elderly through behavioral measures of auditory processing and P300 and controlled for placebo and test–retest effects.

We hypothesized that short-term AT (i.e., ACAT) would generate neurophysiological changes leading to improvements in auditory processing, which is damaged by the degenerative processes of aging. We expected that AT would improve the behavioral performance. We also expected that it would induce earlier P300 peak latencies and increase P300 amplitude at post-test compared to pre-test in the auditory intervention condition alone. Moreover, to assess the effectiveness of ACAT, we investigated the occurrence of test–retest and placebo effects in the study population. Thus, the results can be attributed to ACAT and not the impression that the treatment exerts (positive patient outlooks) or the possibility of learning during the test reapplication.

## Materials and Methods

### Subjects

The present study was approved by the Research Ethics Committee of the Medical School of the Universidade de São Paulo – USP (protocol number: 382/12), and all subjects signed informed consent forms. The subjects were recruited after an analysis of the medical records in a longitudinal study of elderly people conducted at the Department of Physical Therapy, Speech Therapy, and Occupational Therapy at the Medical School of USP in which language, hearing, cognition, and functional capacity were assessed in the elderly volunteers between 2010 and 2013.

The study included 16 subjects (14 women and 2 men) aged 60–78 years with hearing thresholds of up to 40 dB horizontal line (HL) (at 500, 1000, and 2000 Hz); symmetrical hearing configuration; the presence of a V wave evoked with click stimuli during the auditory brainstem response (ABR) (difference of up to 0.2 ms between the ears); and normal scores on the Brazilian version of the Mini-Mental State Examination (MMSE) (Folstein et al., 1975; Brucki et al., 2003) and the Geriatric Depressive Scale (GDS) (Yesavage et al., 1983; Almeida and Almeida, 1999).

Convenience sampling was performed, and more females volunteered to participate in this study. All the subjects complained of decreased speech comprehension and poor performance during at least two auditory skills. None of the patients had a history of psychiatric and/or neurological disorders, used hearing aids, or had previously undergone AT.

### Procedures

The subjects performed an initial assessment (E1) consisting of behavioral and electrophysiological tests and were randomly assigned to two groups. The Passive Control Group (PCG) was composed of eight subjects who did not engage in any activity for 12 weeks and then performed a reevaluation (E2). The Active Control Group (ACG) was composed of eight subjects who participated in a weekly activity (placebo training based on the guidelines proposed by Smith et al., 2009). The weekly activity was watching several 45-min documentaries and answering questions about the videos. The period of video watching was 8 weeks. After 4 weeks (12 weeks after E1), the subjects performed a reevaluation (E2). After E2, all subjects (*N* = 16) received true AT for 8 weeks, and, 4 weeks later, they performed the final evaluation (E3).

**Figure 1:**
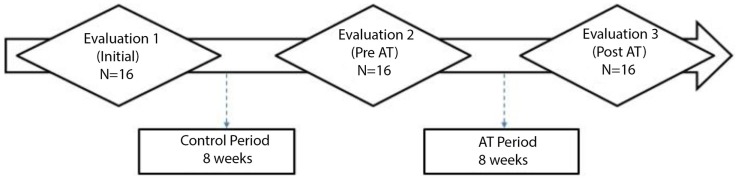
**Study design**.

Figure 1 presents all the stages of the study. The details of the evaluations and AT are described below.

#### Behavioral Assessments

Four different auditory skills were evaluated. Auditory close and dichotic listening to linguistic sounds were evaluated using the Speech-in-Noise and Dichotic Digit tests, respectively, in their adapted Brazilian Portuguese versions (Pereira and Schochat, 2011). Temporal ordering and resolution skills were evaluated using the Pitch Pattern Sequence (Musiek, 1994) and Gap-in-Noise test (Musiek et al., 2005), respectively.

#### Electrophysiologic – P300

A neuroscan electroencephalographic system (Neuroscan Inc., Herndon, VA, USA) model STIM2 was used to record the P300. The potential was evoked using a tone burst stimulus and a complex speech stimulus. In both situations, the stimuli were presented using insert earphones, and the electrodes were positioned at Cz (vertex), A1 (left ear), and A2 (right ear). The subjects were instructed to raise their hand when they detected the rare stimulus. Eye movement control was also performed. The parameters were as follows. In total, 300 artifact-free stimuli were used to obtain the potentials (85% were frequent stimuli, and 15% were rare stimuli). The intensity of the frequent and rare stimuli was 80 dB sound pressure level (SPL). The presentation rate was one stimulus per second. The analysis window was 600 ms with alternating polarity; low-pass filter of 30 Hz; high-pass filter of 1 Hz; and 100 ms duration (plateau of 80 ms and rise/fall of 10 ms). The tone burst was 800 Hz for the frequent stimulus and 1200 Hz for the rare stimulus. The synthesized syllables were/da/and/wa/ (Klatt, 1980) for the frequent and rare stimuli, respectively.

Latency (milliseconds) and amplitude (microvolts) values were analyzed in addition to visual analysis of the waves and images generated for both stimuli. P300 was considered to be the highest positive peak between 250 and 450 ms.

#### Acoustically Controlled Auditory Training

The AT used in this study followed the characteristics proposed by Musiek and Schochat (1998). It was conducted in a soundproof booth for 8 weeks with one 50-min session per week.

The impaired skills detected at E2 were trained, and at the end of each session, 10 min were reserved for guidance regarding communication strategies and the performance of home-based activities (approximately 15 min, three times a week). Each session was planned according to the individual’s performance during the previous session while attempting to maintain a success/error rate of approximately 70/30% (Musiek and Schochat, 1998) and stimulating at least three hearing skills. Verbal and non-verbal stimuli were used. Where possible, both perceptual activities (discrimination of monosyllabic words and compressed disyllabic words, comprehension of sentences in the presence of different types of noise and competitive speech, discrimination and ordering of pure tones, and perception of gaps) and cognitive skills, such as working memory (discrimination of five words among noise and repetition in reverse order of the sequence heard), sensory integration by the aggregation of visual tasks (identification of written sentences), and motor tasks (e.g., pointing to figures corresponding to descriptions heard in the right ear using the left hand) were used in the training. Moreover, we attempted to use a motivating approach, considering the age, preferences, and habits of each individual.

#### Statistical Analysis

To compare the average results of the tests in both groups and both ears and the test scores for the three study periods evaluated, multivariate analysis of variance (MANOVA) and MANOVA with repeated measures (repeated-measures MANOVA) were applied, respectively (Dancey and Reidy, 2013). To complement the descriptive analyses, we used confidence intervals (CI) to assess the extent to which the average could vary within a certain confidence probability. The CI established for our data analysis was 95% with a significance level (*p*) of 0.05 (5%).

## Results

### Placebo and test–retest effects

**Figure 2:**
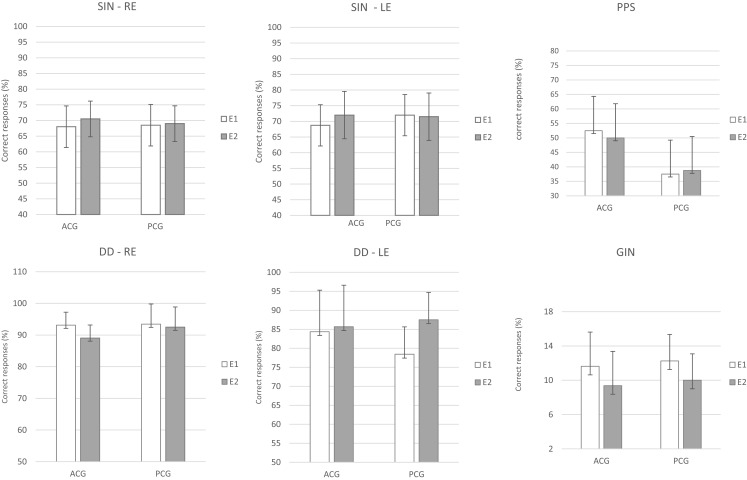
**Mean results and error bars indicating the 95% CIs between E1 and E2 for the behavioral tests**. SIN, speech-in-noise; DD, dichotic digits; GIN, gap-in-noise; PPS, pitch pattern sequence; RE, right ear; LE, left ear; E1, evaluation 1 (initial); E2, evaluation 2 (pre-ACAT); ACG, active control group; PCG, passive control group.

**Figure 3:**
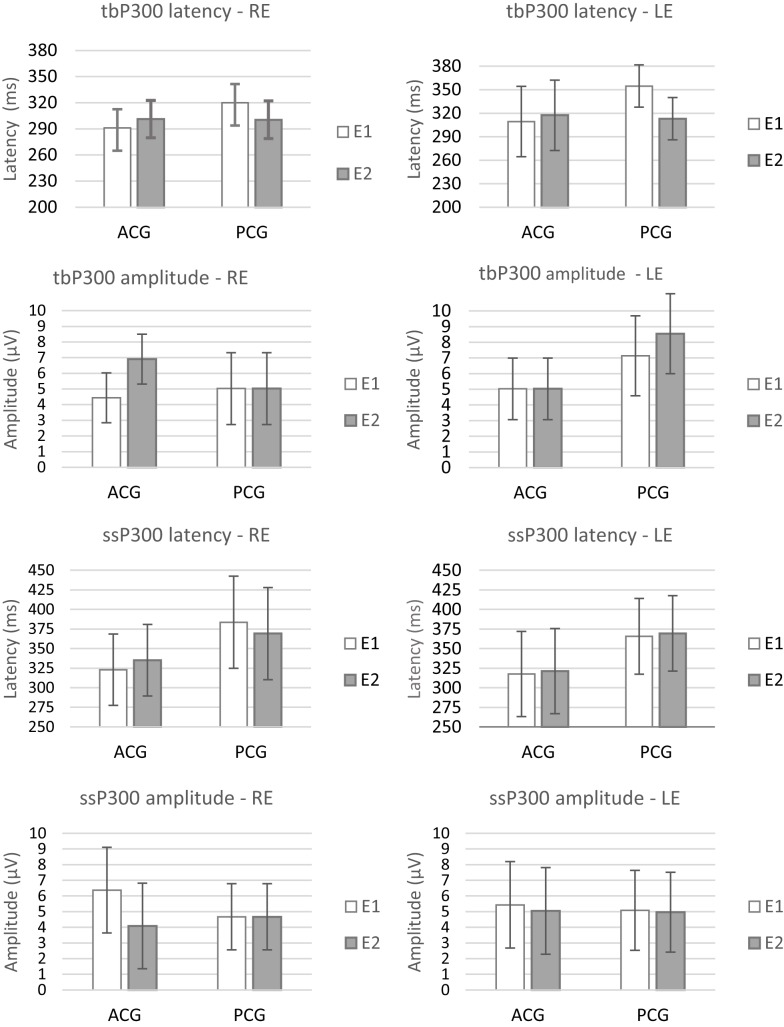
**Mean results and error bars indicating the 95% CIs between E1 and E2 for the latency and amplitude of P300 RE, right ear; LE, left ear; E1, evaluation 1 (initial); E2, evaluation 2 (pre-ACAT); ACG, active control group; PCG, passive control group**.

The repeated-measures MANOVA indicated no significant differences between the two assessments (E1 and E2) or between the groups (PCG and ACG) for the behavioral [*F*(9.6) = 0.36, *p* = 0.92, Wilks λ = 0.65] or electrophysiological tests [*F*(8.7) = 2.11, *p* = 0.17, Wilks λ = 0.29]. The existence of a significant overlap in the 95% CIs for all the tests in the same period (E1 and E2) and between the groups indicated the absence of a real effect in the E2 period, i.e., no placebo or test–retest effects occurred. Figures 2 and 3 present the CIs for the behavioral and electrophysiological tests, respectively, at assessments E1 and E2.

### Effect of ACAT

Considering the lack of a significant difference between E1 and E2 and between the two groups, the effect of ACAT was assessed after combining the two initial groups into one group (*n* = 16), which was designated the Auditory Training Group (ATG). The repeated-measures MANOVA revealed a multivariate difference between E2 (pre-ACAT) and E3 (post-ACAT) for the behavioral tests [*F*(9.7) = 4.95, *p* = 0.02*, partial η^2^ = 0.86, Wilks λ = 0.13]. To assess the relative contribution of each behavioral test to the multivariate differences, univariate analyses were performed (Table 1). Significant differences between E2 and E3 were observed for the following variables: SIN RE (*p* < 0.001***); SIN LE (*p* = 0.002**); DD LE (*p* = 0.008**); GIN (*p* = 0.006**); and PPS (*p* < 0.001***). The variable DD was almost significant (*p* = 0.08^#^). Furthermore, we verified that the most difficult task for elderly people was temporal processing, which was reflected by the values obtained in the PPS, compared to other skills measured.

**Table 1:** **Mean values, SD, and *p*-values for the performance of elderly people (*n* = 16) during the behavioral tests at E2 (pre-ACAT) and E3 (post-ACAT)**.

		E2		E3		*p*-Value
		Mean	SD	Mean	SD	
GIN (ms)		9.69	3.94	7.75	2.57	0.006**
PPS (%)		44.38	16.01	62.81	15.16	0.001**
DD (%)	RE	90.78	8.30	94.23	5.37	0.08^a^
	LE	86.59	9.22	90.78	5.61	0.008**
SIN (%)	RE	69.75	7.30	79.50	7.28	<0.001***
	LE	71.75	9.63	81.25	8.23	0.002**

SIN, speech-in-noise; DD, dichotic digits; GIN, gap-in-noise; PPS, pitch pattern sequence; RE, right ear; LE, left ear; E2, evaluation 2 (pre-ACAT); E3, evaluation 3 (post-ACAT); SD, standard deviation; **0.05 or less; ***0.01 or less;

*^a^trend toward significance*.

**Figure 4:**
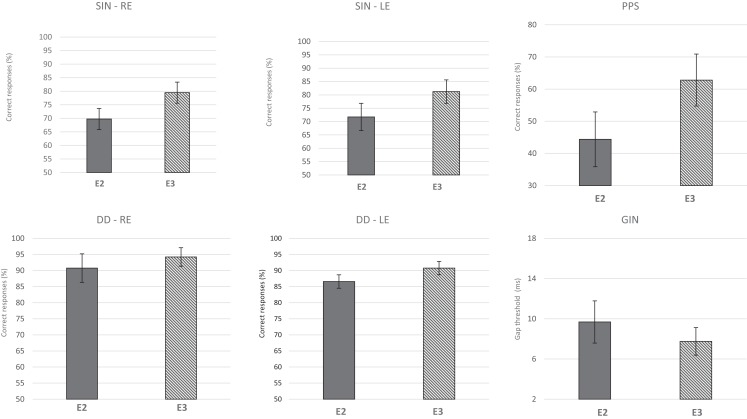
**Mean results and error bars indicating the 95% CIs of E2 and E3 for the behavioral tests**. SIN, speech in noise; DD, dichotic digits; GIN, gap-in-noise; PPS, pitch pattern sequence; RE, right ear; LE, left ear; E2, evaluation 2 (pre-ACAT); E3, evaluation 3 (post-ACAT).

Figure 4 presents the CIs for the behavioral tests at assessments E2 and E3. These results indicate that ACAT improved all of the auditory skills evaluated. The repeated-measures MANOVA indicated no significant differences for the P300 variables (Table 2) with either the tone burst stimulus or the speech-sound stimulus between the periods E2 and E3 [*F*(8.8) = 0.61, *p* = 0.74, Wilks λ = 0.62].

**Table 2:** **Mean values, SDs, and *p*-values for the latency (milliseconds) and amplitude (microvolts) measures of P300 using tone burst and speech-sound stimuli in elderly people (*n* = 16) at E2 (pre-ACAT) and E3 (post-ACAT)**.

		E2		E3	
		Mean	SD	Mean	SD
tbP300 latency	RE	300.90	27.39	292.00	27.64
	LE	315.23	34.41	310.01	62.05
tbP300 amplitude	RE	6.21	3.00	6.65	2.55
	LE	6.79	3.72	7.53	2.96
ssP300 latency	RE	352.10	77.07	332.46	70.12
	LE	345.38	66.32	328.17	54.30
ssP300 amplitude	RE	4.38	2.71	5.22	3.70
	LE	5.00	3.26	6.21	3.26
*p*-Value		0.74			

*tbP300, tone burst P300; ssP300, speech sound P300; RE, right ear; LE, left ear; E2, evaluation 2 (pre-ACAT); E3, evaluation 3 (post-ACAT) SD, standard deviation*.

**Figure 5:**
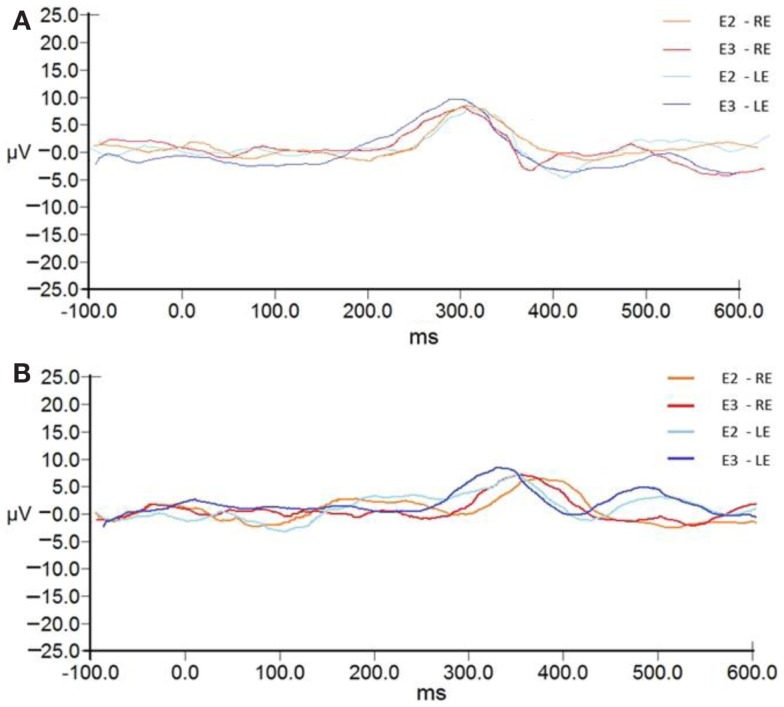
**P300 group averages pre- and post-ACAT for both stimuli**. **(A)** presents the waves to tone bursts. **(B)** presents the waves to the speech stimulus. Note later latencies and lower amplitudes for the stimulation of speech. RE, right ear; LE, left ear; E2, evaluation 2 (pre-AT); E3, evaluation 3 (post-AT).

**Figure 6:**
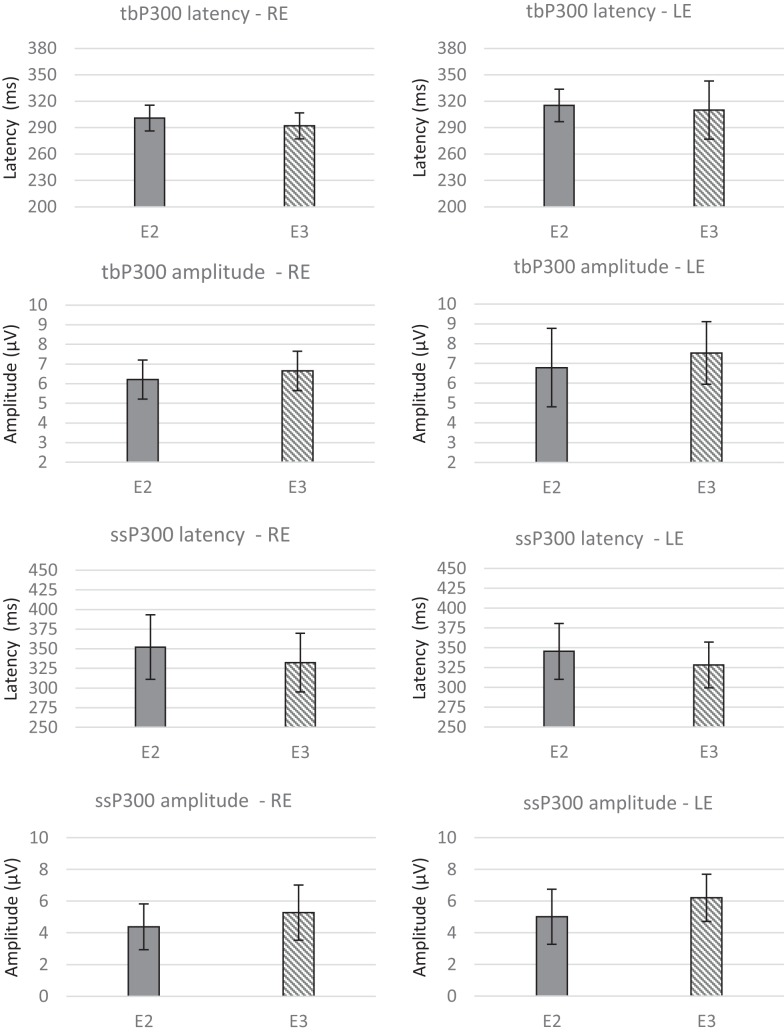
**Mean values and error bars indicating the 95% CIs between E2 and E3 for the electrophysiological tests**. RE, right ear; LE, left ear; E2, evaluation 2 (pre-ACAT); E3, evaluation 3 (post-ACAT).

**Figure 7:**
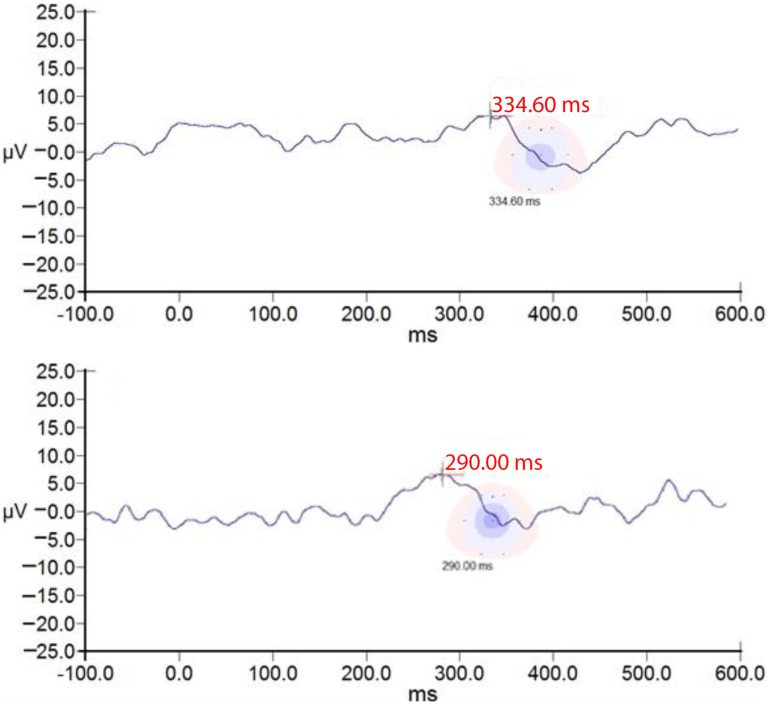
**ssP300 RE morphology of one subject at E2 and E3**. ssP300 RE of one subject at E2 **(A)** and E3 **(B)**. Note better latency, morphology of the ssP300 wave after auditory training.

The P300 group averages pre- and post-ACAT for both stimuli are presented in Figure 5. Figure 6 presents the CIs for the electrophysiological tests at E2 and E3. No significant differences were observed between the types of stimuli used for assessing ACAT [*F*(4.27) = 0.18, *p* = 0.94, Wilks λ = 0.97]. Although there were no significant differences between the evaluations, it is important to note that in several subjects receiving ACAT, we observed better latencies, amplitudes, and waveform morphologies of the P300 waves (Figure 7).

## Discussion

### Placebo and test–retest effects

The main objective of this study was to evaluate the effectiveness of ACAT in elderly individuals with two or more impaired auditory processing skills. For this purpose, we initially investigated the occurrence of test–retest and placebo effects. No evidence of these effects (test–retest and placebo) was found for any of the tests (behavioral or electrophysiological) evaluated (Figures 2 and 3). This result indicates that the tasks performed during this period did not produce different behaviors from the ones presented initially. This finding confirms the results of previous studies on neuroplasticity, indicating that increases in coordination and synchronization of the neural responses occur only through “learning-driven training” and consequently is “a primary determinant of the feed-forward power of any plastically strengthening cortical process” (Merzenich et al., 2014). This finding indicates that the changes observed after the interventions are attributed exclusively to the proposed ACAT (Anderson et al., 2013a; Ferguson et al., 2014).

Regarding auditory processing abilities, we observed that the most difficult task for elderly people was temporal processing as reflected by the values obtained in the PPS (E2/pre-ACAT). According to Tallal and Newcombe (1978), there is a direct association between temporal acoustic perception and speech perception. Therefore, the temporal-processing deficit due to aging may contribute to the impairment of speech perception. In other words, the accuracy of this coding is impaired by aging and directly affects speech understanding.

Temporal processing difficulties among elderly people have been corroborated by several studies (Gordon-Salant and Fitzgibbons, 1999; Phillips et al., 2000; Parra et al., 2004; Martin and Jerger, 2005; Liporaci and Frota, 2010). In addition, other studies (Bellis and Wilber, 2001; Azzolini and Ferreira, 2010; Hennig et al., 2012) have demonstrated that the performance of elderly people on the same test used in the present study (PPS) was very similar to the performance of young adults, although the responses were obtained by humming (not naming). One possible explanation is related to the decreased function of the corpus callosum. When this test is performed through the acquisition of a verbal response that requires naming, the auditory information of the tonal pattern is detected and recognized by the right hemisphere and is then conducted (via the corpus callosum) to the left hemisphere. Then, these stimuli are associated with their linguistic representations and the organization of verbal responses (Musiek and Pinheiro, 1987). When the same test is performed by humming, inter-hemispheric transfer via the corpus callosum is not required.

Other studies using objective techniques, such as functional magnetic resonance imaging (Salat et al., 1997; Silver et al., 1997; Gootjes et al., 2006), have revealed anatomical changes due to aging, including decreased mass in specific regions and decreased fiber myelination in the corpus callosum. Therefore, our findings reinforce the hypothesis that the aging process impairs the function of the corpus callosum.

### Effect of acoustical controlled auditory training

Specifically, we demonstrated that short-term training-induced neural plasticity in older adults in some aspects of auditory processing. After directed stimulation using ACAT, a significant improvement was observed in all auditory skills, which are observed through behavioral assessment tests (Table 1; Figure 4). These findings are supported by other studies that report the occurrence of neuroplasticity even during conditions of aging (Swain and Richard, 1993; Merzenich and DeCharms, 1996; Kilgard et al., 2001; Weinberger, 2004; Gilbert et al., 2009; Merzenich et al., 2013, 2014). Neuroplasticity is the intrinsic property of the nervous system that allows the development of structural changes in response to experiences and environmental changes (Pascual-Leone et al., 2005). Therefore, intense auditory stimulation during AT modifies the function of the auditory system leading to positive behavioral changes (Musiek et al., 2002; Fritz et al., 2005; Song et al., 2011).

The optimal intensity and duration of AT remain unknown (Molloy et al., 2012). In most studies (Tremblay and Kraus, 2002; Burk et al., 2006; Burk and Humes, 2008; Smith et al., 2009; Anderson et al., 2013a, 2014; Ferguson et al., 2014), the frequency and duration of AT differed from our study and the sessions were performed daily or more than once per week (Anderson and Kraus, 2013). In addition to the intensity, frequency, and duration of AT, previous studies performing AT have also differed in their methods, stimuli, and other variables (home- or lab-based and tone, phoneme, word, or sentence-based training). These differences make comparisons between studies difficult. However, we highlight that the ACAT proposed in our study significantly improved the auditory skills of elderly people even when performing the exercises only once per week for 8 weeks. Despite the promising results described in the present study using behavioral assessments, similar pre- and post-ACAT changes were not achieved with the electrophysiological assessments of P300 with tone burst or speech stimuli (Table 2; Figure 6).

Several studies have demonstrated the effectiveness of AEPs (short, medium, and long latency) to monitor the neurophysiological changes arising from AT using tone burst (Jirsa, 1992; Tremblay and Kraus, 2002; Gil, 2006; Musiek et al., 2007; Alonso and Schochat, 2009) and speech stimuli (Tremblay et al., 2001; Tremblay and Kraus, 2002; Alonso, 2011; Anderson et al., 2013a,b). However, the electrophysiological evaluation of P300 has some peculiarities in relation to other electrophysiological tests. P300 can be influenced by the test parameters, including the type of stimulus, inter-stimulus interval, type of task, cognitive factors (attention and memory), hormonal factors, and other factors (Patterson et al., 1988; Kügler et al., 1993; Polich, 1996; Hirayasu et al., 2000). These parameters may be altered in elderly people. Therefore, we believe that the possible significant effect of the two stimuli used for the P300 potential depends on many other factors in addition to the factors that govern auditory processing.

One of the factors that causes social isolation among elderly people is the well-known complaint that “I hear, but I don’t understand.” Therefore, ACAT should be considered for the management of communication difficulties in older adults and may mitigate some of the psychosocial sequelae that can exacerbate aging effects, such as depression and social isolation.

Considering the difficulties experienced by the subjects regarding auditory processing and pattern changes during post-stimulation auditory processing, we believe that the proposed ACAT can promote changes in behavioral performance in elderly people for all the auditory skills investigated. ACAT was an effective method for auditory rehabilitation in elderly people with APD. Although the present study did not find any evidence of neuroplasticity (P300), we encourage the use of other electrophysiological measures to monitor neurophysiological changes during the aging process. In addition, other studies should evaluate the maintenance of treatment effects over time, including self-assessment and quality of life questionnaires, to confirm the incorporation of these improved auditory skills into daily life.

In conclusion, this study demonstrates that ACAT (short-term training) can improve auditory processing even during the degenerative processes caused by aging.

## Conflict of Interest Statement

The authors declare that the research was conducted in the absence of any commercial or financial relationships that could be construed as a potential conflict of interest.
